# Randomized controlled trial of the Tobacco Tactics website versus 1-800-QUIT-NOW telephone line among Operating Engineers

**DOI:** 10.1186/1617-9625-12-S1-A13

**Published:** 2014-06-06

**Authors:** Seung Hee Choi, Andrea H Waltje, David L Ronis, Devon Noonan, Oisaeng Hong, Caroline Richardson, John D Meeker, Sonia A Duffy

**Affiliations:** 1 School of Nursing, University of Michigan, Ann Arbor, Michigan, 48105, USA; 2 School of Nursing, Duke University, Durham, North Carolina, 27708, USA; 3 Department of Community Health Systems, University of California, San Francisco, California, 94143, USA; 4 Ann Arbor VA Center for Clinical Management Research, Health Services Research and Development, Ann Arbor, Michigan, 48105, USA; 5 Department of Family Medicine, University of Michigan, Ann Arbor, Michigan, 48105, USA; 6 School of Public Health, University of Michigan, Ann Arbor, Michigan, 48105, USA; 7 Department of Otolaryngology, University of Michigan, Ann Arbor, Michigan, 48105, USA; 8 Department of Psychiatry, University of Michigan, Ann Arbor, Michigan, 48105, USA

## Background

The purpose of this study was to evaluate the efficacy and usage of the Tobacco Tactics website compared to the 1-800-QUIT-NOW telephone line among Operating Engineers (heavy equipment operators).

## Materials and methods

Smokers attending workplace safety training groups were randomized to either the Tobacco Tactics website with nurse phone counseling and access to nicotine replacement therapy (NRT) or to the 1-800-QUIT-NOW telephone line which provided an equal number of phone calls and NRT. Participating Operating Engineers completed a baseline survey as well as mailed surveys at 30-days and 6-months. Urinary cotinine tests were used to verify 6-month smoking status. The outcomes were compared using χ2 tests, t-tests, mixed models, generalized mixed models, and logistic regression models.

## Results

Compared to participants in the 1-800-QUIT-NOW group, significantly more of those in the Tobacco Tactics website group participated in the intervention, received phone calls and found the intervention helpful (p<0.05). Seventy percent of the website group received NRT compared to 5.1% of the quitline group (p<0.001). At 30-day follow-up, the Tobacco Tactics website group showed significantly higher quit rates (26.9%) than the 1-800-QUIT-NOW group (7.7%) (p<0.05), but this difference was no longer significant at 6-month follow-up. There were significantly more positive changes in harm reduction measures (quit attempts, number of cigarettes smoked per day, and nicotine dependence) at both 30-day and 6-month follow-up in the Tobacco Tactics website group compared to the 1-800-QUIT-NOW group (p<0.05).

## Conclusions

The Tobacco Tactics website showed higher efficacy and reach than the 1-800-QUIT-NOW intervention. Longer counseling sessions may be needed to improve 6-month cessation rates. This intervention has the potential to reduce morbidity and mortality among Operating Engineers.

